# Suppression of the pelo protein by *Wolbachia* and its effect on dengue virus in *Aedes aegypti*

**DOI:** 10.1371/journal.pntd.0006405

**Published:** 2018-04-11

**Authors:** Sultan Asad, Mazhar Hussain, Leon Hugo, Solomon Osei-Amo, Guangmei Zhang, Daniel Watterson, Sassan Asgari

**Affiliations:** 1 Australian Infectious Disease Research Centre, School of Biological Sciences, The University of Queensland, Brisbane Australia; 2 QIMR Berghofer Medical Research Institute, Herston, Australia; 3 School of Chemistry and Molecular Biosciences, The University of Queensland, Brisbane Australia; University of Florida, UNITED STATES

## Abstract

The endosymbiont *Wolbachia* is known to block replication of several important arboviruses, including dengue virus (DENV), in the mosquito vector *Aedes aegypti*. So far, the exact mechanism of this viral inhibition is not fully understood. A recent study in *Drosophila melanogaster* has demonstrated an interaction between the *pelo* gene and Drosophila C virus. In this study, we explored the possible involvement of the pelo protein, that is involved in protein translation, in *Wolbachia*-mediated antiviral response and mosquito-DENV interaction. We found that pelo is upregulated during DENV replication and its silencing leads to reduced DENV virion production suggesting that it facilities DENV replication. However, in the presence of *Wolbachia*, specifically in female mosquitoes, the pelo protein is downregulated and its subcellular localization is altered, which could contribute to reduction in DENV replication in *Ae*. *aegypti*. In addition, we show that the microRNA aae-miR-2940-5p, whose abundance is highly enriched in *Wolbachia*-infected mosquitoes, might mediate regulation of pelo. Our data reveals identification of pelo as a host factor that is positively involved in DENV replication, and its suppression in the presence of *Wolbachia* may contribute to virus blocking exhibited by the endosymbiont.

## Author summary

Dengue infection along with its related disease conditions poses a significant threat to human populations. The pathogen responsible for this infection is dengue virus (DENV), which is primarily transmitted to humans through the bites of *Ae*. *aegypti* mosquitoes. Unavailability of vaccines has recently sparked research endeavours aimed at vector control. To date, *Wolbachia* as an endosymbiotic bacterium, has shown promises as a novel biocontrol agent to restrict DENV replication in mosquitoes through a mechanism that is still elusive. In this study, we investigated the role of pelo in *Wolbachia*-mosquito-DENV interactions. We found that the pelo protein is upregulated upon DENV infection facilitating its replication, but *Wolbachia* infection leads to the suppression of the protein that may contribute to inhibition of DENV replication. Our findings provide novel insights into the role of the pelo protein in *Wolbachia*-mediated antiviral effect against DENV in *Ae*. *aegypti* mosquitoes.

## Introduction

Dengue virus (DENV) is one of the major medically important arboviruses [1, 2]. It belongs to the *Flaviviridae* family that comprises lipid-enveloped, positive-sense single stranded RNA viruses [3]. DENV is classified into four antigenically distinct but closely related serotypes, designated as DENV-1 to DENV-4. The bite of an infected female of either *Aedes aegypti* or *Aedes albopictus* is the common mode of DENV transmission to humans [4]. Infected humans may suffer from dengue fever (DF), dengue shock syndrome (DSS) or dengue haemorrhagic fever (DHF), leading to fatality [5, 6]. Currently, there is no specific therapy or effective vaccine available and the treatments available are palliative in nature. Despite substantial efforts to control DENV through vector control, it is still geographically expanding and alternative vector control strategies and therapeutic options are urgently needed [7]. One such strategy involves the use of a bacterial endosymbiont *Wolbachia* in transinfected *Ae*. *aegypti* mosquitoes which limits DENV replication [8, 9].

*Wolbachia* is an endosymbiotic, vertically transmitted bacterium that appears to have infected more than 40% of insect species in addition to other terrestrial arthropods [10]. However, it is not a natural symbiont of *Ae*. *aegypti*, which is the primary vector of DENV. Recently, several different types of *Wolbachia* strains have been successfully introduced into *Ae*. *aegypti* mosquitoes, among which *Wolbachia w*Mel-Pop-CLA (Pop) and *w*Mel strains are the most promising ones [11, 12]. Both strains produce cytoplasmic incompatibility (CI), which facilitates replacement of the wild populations through this reproductive manipulation [13, 14]. Furthermore, introduction of *Wolbachia* has induced viral resistance in *Ae*. *aegypti* mosquitoes to a variety of arboviruses including dengue, Zika, West Nile and chikungunya viruses [11, 15–17]. However, the exact mechanism which causes this antiviral effect is not fully understood. Despite a small number of studies that have elucidated the potential role of microRNAs (miRNAs) [18, 19], reactive oxygen species (ROS) [20, 21], and competition for resources [22] in *Wolbachia*-mediated antiviral response, the fundamental molecular mechanism(s) underlying virus blocking are yet to be explored. One of the possible strategies to discover molecular interactions between DENV-*Wolbachia*-*Ae*. *aegypti* is to find host factors that facilitate replication of arboviruses and examine their relative abundance in the presence of *Wolbachia* infection. A recent study in *Drosophila melanogaster* has identified the protein pelo as an important host factor facilitating efficient replication of Drosophila C virus (DCV) by improving access of the viral genome to ribosomes leading to increased synthesis of viral structural proteins [23].

Pelo is an evolutionary conserved protein which plays an important role in the regulation of *D*. *melanogaster* germ cell meiosis. In the pelo mutant males of *D*. *melanogaster*, cell cycle in spermatogenesis is arrested at the late prophase stage [24, 25]. In contrast, in mutant female flies, mitotic division during oogenesis is affected along with impaired development of the eyes [26]. Deletion of pelo’s homolog, (Dom34, Duplication Of Multilocus Region) in yeast *Saccharomyces cerevisiae* led to the build-up of free ribosomes and decline in the number of polysomes suggesting its involvement in translational regulation [27].

In this study, we aimed to characterize the expression of *Ae*. *aegypti’*s pelo protein in response to *Wolbachia* and DENV infection. We found that the pelo protein facilitates DENV maturation/secretion and *Wolbachia* suppresses the protein which may contribute to the inhibition of DENV in *Wolbachia*-infected cells. In addition, we explored the subcellular localization of the pelo protein in response to *Wolbachia* and DENV infection. Furthermore, we demonstrate an indirect involvement of the miRNA aae-miR-2940-5p in regulating pelo in response to *Wolbachia* infection.

## Methods

### Mosquitoes, flies and cell lines

All mosquitoes used in this study for *Wolbachia* comparisons were previously produced by McMeniman et al. (2008) by infecting *Ae*. *aegypti* mosquitoes with *w*MelPop-CLA strain of *Wolbachia* (Pop). Uninfected mosquitoes were generated by tetracycline (Tet) treatment to ensure the complete removal of *Wolbachia* infection [13]. After confirmation of *Wolbachia* removal, the Tet mosquitoes were no longer under tetracycline treatment. To ensure the availability of functional gut microflora the Tet mosquitoes were allowed to feed for two generations on water used to raise natural mosquito larvae [13]. *Ae*. *aegypti* mosquitoes used for dsRNA experiments were obtained from a colony established from adults collected from Innisfail, north Queensland, and maintained at the QIMR Berghofer Medical Research Institute. *w*1118 fly line stably infected with *w*MelPop (Pop) and the tetracycline cured line (Tet) were generated by Min et al. (1997) [14]. *Ae*. *aegypti* Aag2 and Pop cell lines previously described by Frentiu et al. (2010) were maintained in 1:1 Mitsuhashi-Maramorosch and Schneider’s insect medium (Invitrogen) supplemented with 10% FBS (Bovogen Biologicals) [28]. For microRNA direct interaction analysis, *Spodoptera frugiperda* Sf9 cells were used which were maintained in Sf900II medium (Life Technologies). All the above-mentioned cell lines were kept at 28°C and passaged after every 3–4 days.

### RT-PCR and qPCR analyses

Total RNA was extracted from either cell lines, mosquitoes or flies using Qiazol (Qiagen) and subsequently treated with Turbo DNase (Ambion) according to the manufacturer’s guidelines. Total RNA (1000ng or 200 ng for tissues with less RNA) was used to make cDNA using Superscript III (Invitrogen) using either oligo(dT) primer for cellular genes or DENV-qR primer to amplify the DENV genomic RNA. The *pelo* transcript sequence (XM_001658653.1) was retrieved from NCBI in order to design primers. The full-length *pelo* gene was amplified by Taq DNA polymerase (New England Biolabs) using 1μl of cDNA in 25μl reaction together with *pelo*-specific primers (all primer sequences are listed in S1 Table).

The cDNA reaction was diluted in 1:5 ratio with water, from which 2μl was used for qPCR reactions in which gene-specific primers were utilized to amplify the target genes, using QuantiFast SYBR Green (Qiagen) in a Rotorgene thermocycler (Qiagen). For mosquito samples, ribosomal protein S17 (*RPS17*; AY927787.2) transcripts were used for normalization of RNA templates, while ribosomal protein L32 (*RPL32*; NM_079843) was used for normalization in the case of *D*. *melanogaster*. Each qPCR reaction was performed in two technical replicates each with at least three biological replicates. All qPCR data were normalized with Qiagen analysis templates and were further analysed by Prism 6.0. The qPCR cycling profile included 95°C for 5 min for the initial denaturation and hot start Taq activation, and 40 cycles of 95°C for 10 sec, 60°C for 15 sec and 72°C for 20 sec. Melt curve analysis was carried out on default settings of the Rotorgene machine to check the specificity of the qPCR products. Unpaired t-test was used to compare differences between two individual groups while One-way ANOVA with Tukey’s PostHoc test was performed to find differences between more than two groups of data.

### Nuclear and cytoplasmic fractionation

Nuclear and cytoplasmic fractions of Aag2 and Pop cells were separated using the PARIS kit (Ambion) according to manufacturer's instructions. Briefly, cells were washed with 1×PBS, centrifuged at 200 ×g at 4°C, followed by resuspension of the cell pellet in 300μl ice-cold cell fractionation buffer, incubated on ice for 10 min, centrifuged at 300 ×g at 4°C for 5 min. The supernatant was collected as the cytoplasmic fraction, and the pellet was washed five times with 200μl ice-cold cell fractionation buffer to ensure removal of traces of the cytoplasmic fraction and considered as the nuclear fraction. The pellet containing the nuclear fraction was resuspended in 300μl of cell disruption buffer. 300μl of 4×SDS-PAGE buffer was added to both nuclear and cytoplasmic fractions.

### Western blot analysis

RIPA buffer (Sigma) was used to get total cell lysates from cells and mosquitoes. Briefly, samples were homogenised with the help of 2mm glass beads using a TissueLyser II (Qiagen) at 30 frequency for 90 sec. The lysate was then centrifuged at 4°C at full speed for 5 min and supernatant was collected for protein analysis. Relative protein concentrations were determined by measuring absorption at 280 nm (Epoch).

Protein samples were run on 10% polyacrylamide gels, transferred to nitrocellulose membrane and then blocked with 5% skimmed milk for 1 hr at room temperature with continuous shaking. After 3 times washing with 1×TBST, membrane was incubated with either anti-pelo (raised in rabbit) [23], anti-GAPDH (Sigma, raised in rabbit), anti-HSP70 (Sigma, raised in rabbit) or anti-DENV envelope protein antibody (Abcam, raised in rabbit) overnight at 4°C on shaker. For secondary antibody incubation, the blot was washed three times with 1×TBST and then incubated with secondary antibodies either conjugated with alkaline phosphatase (Sigma, anti-rabbit raised in goat) or infrared detection probe (IRDye 800CW goat anti-human LI-COR). After 1 h of incubation, the blot was again washed three times with 1×TBST and then it was further incubated with 5-bromo-4-chloro-3-indolyl-phosphate/nitro blue tetrazolium (BCIP/NBT) ready to use solution (Thermoscientific) or scanned with an Odyssey imager (LI-COR Biosciences).

### Oversupply and inhibition of aae-miR-2940-5p

Aag2 cells were plated in a 12-well plate and transfected with 100 μM of artificially synthesized mimic or inhibitor of either aae-miR-2940-5p or non-specific control mimic/inhibitor (GenePharma) (sequences in S1 Table). Cellfectin was used as the transfection reagent (Invitrogen). Cells were collected 72 h post-transfection for downstream analyses.

### Northern blot analysis

20 μg of total RNA was run on a 15% urea denaturing polyacrylamide gel, electro-blotted onto a nylon membrane by a semi-dry Western blotting apparatus (Bio-Rad), and UV cross-linked. The blot was blocked in ExpressHyb hybridization solution (Clonetech) for 1h in a rotary oven at 50°C. Oligonucleotides (20–23 mer) reverse complementary to aae-miR-2940-5p and U6 sequences were labelled with [α-32P]-dATP using terminal nucleotide transferase kit (New England Biolabs). Probe hybridization was carried out in ExpressHyb hybridization buffer (Clonetech) and washes were done under stringent conditions (3× SSC + 5% SDS twice and 1× SSC + 1% SDS twice) at 50°C. The blot was exposed to a phosphorimager screen overnight, and signals were detected using a Storm phosphorimager scanner (Amersham). To reprobe with U6, the blot was stripped to remove the aae-miR-2940-5p probe with boiling in 0.1% SDS twice for 30 min each time on a shaker. The stripped blot was checked for any radioactive signal by scanning it with Storm phosphorimager scanner.

### RNAi-mediated gene silencing

To knockdown the transcript levels of the *pelo* gene for functional analyses during DENV replication, primers were designed to amplify a 552 bp fragment from the *pelo* gene containing the T7 promoter sequences at both ends. The MEGAscript T7 Transcription kit (Ambion) was used according to the manufacturer’s instructions to produce dsRNA targeting the *pelo* transcripts. The same approach was used to synthesize dsRNA against *Ae*. *aegypti Argonaut 1* (*Ago1*; XP_001662554), *Ago2* (FJ979880.1), and *Green fluorescent protein* (*GFP*; X83960). To knockdown genes, cells in each well were double transfected with 5 and 2μg of dsRNA, respectively. As control, dsGFP RNA was used.

For *in vivo* silencing of *pelo*, 3-day old females were injected with 200 nL of dsRNA (dspelo or dsGFP as control) or filtered PBS. Capillary needles (Drummond) were pulled using a Narishige capillary puller using single step program at 86°C. Mosquitoes were aspirated from the cups, anaesthetized with CO_2_, then placed onto a petri dish on ice under a stereo-dissecting microscope. Females were intra-thoracically injected with 200 nL of appropriate treatment solution (stock concentration 4μg/μl in the case of dsRNAs) using a Nanoject III (Drummond) microinjector at a flow rate of 100 mL/sec. Between 102–104 females were injected for each treatment. Injected mosquitoes were placed into 750ml plastic gauze top containers, provided with 10% sugar solution on cotton pledgets. Injected mosquitoes were maintained in an Environmental Growth Chamber (Panasonic) maintained at 28°C, 75% RH and 12:12 hour day:night and fading regulated by a 6 step light cycle.

### Virus infection and plaque assay

For *in vitro* virus infection experiments, *Ae*. *aegypti* Aa20 cells [29] were seeded at the density of 3×10^5^ cells per well in 12-well plates. After settlement of cells, they were double transfected with dsRNA against the target gene or the control. After 6 h post-secondary transfection, they were infected with DENV-2 (New Guinea strain) at the multiplicity of infection (MOI) indicated in the text. Media were collected 72 h after infection for plaque assay and the cells were subjected to RNA extraction to check the DENV genomic RNA levels by RT-qPCR.

For *in vivo* infection, 48 h after injection of mosquitoes with dsRNAs, mosquitoes were offered the opportunity to feed on a mixture of DENV-2 (QML16) strain virus supernatant in defibrinated sheep blood at a titre of 10^7^ pfu/ml using an artificial membrane feeding apparatus with dried bovine caecum lining as the membrane. A second group of the dsRNA injected mosquitoes was intra-thoracically microinjected with 50 nL of the DENV-2 stock (10^7^ pfu/ml) using the Nanoject III microinjector at a flow rate of 25 nL/sec while maintained on ice. Following DENV injection, mosquitoes were returned to the 750 ml plastic container for maintenance in the environmental growth chamber and collected 7 days post virus injection. Mosquito lysates were subsequently prepared by homogenizing the body of each mosquito in 500 μl of OptiMEM medium (Sigma) containing 2.5% FBS followed by centrifugation at 5900 ×g for 5 min, at 4°C. The supernatant was collected and used for virus titration and RNA extraction in order to confirm the *pelo* knock down.

For plaque assays, Vero cells monolayers previously seeded in a 96-well plate were inoculated with serial dilutions of media or mosquito lysates (10^0^,10^−1^,10^−2^, 10^−3^) collected from experiments in duplicates. Plates were first incubated at room temperature on a rocker for 1 h and then incubated at 37°C for an extra hour. Subsequently, media were removed and cells were overlayed with 1.5% carboxymethyl cellulose (CMC) and 2.5% FBS in OptiMEM medium. Cells were then incubated for 72 h at 37°C and 5% CO_2_. Subsequently, the overlay was removed and the cells were fixed with 80% ice-cold acetone in 1×PBS for 20 min at -20°C, and then air dried overnight. Then cells were blocked with 5% skimmed milk in 1×PBST at 37°C for 30 min. This was followed by incubating cells with the primary antibody against DENV-2-Envelope (human) in 1:1000 dilution in 0.1% skimmed milk in 1×PBST for 2 h at 37°C as described previously [30]. After that, plates were washed three times with 1×PBST and then incubated with the secondary antibody (IRDye 800CW goat anti-human LI-COR) for 1 h at 37°C. Plates were washed three times with 1×PBST and dried as above and scanned on the Odyssey imager (LI-COR Biosciences) at 41μM resolution. Plaques were counted and viral titre was calculated accordingly. Plaque numbers obtained for compound titration were performed in triplicates.

## Results

### Pelo is ubiquitously expressed in mosquito tissues

**Fig 1:**
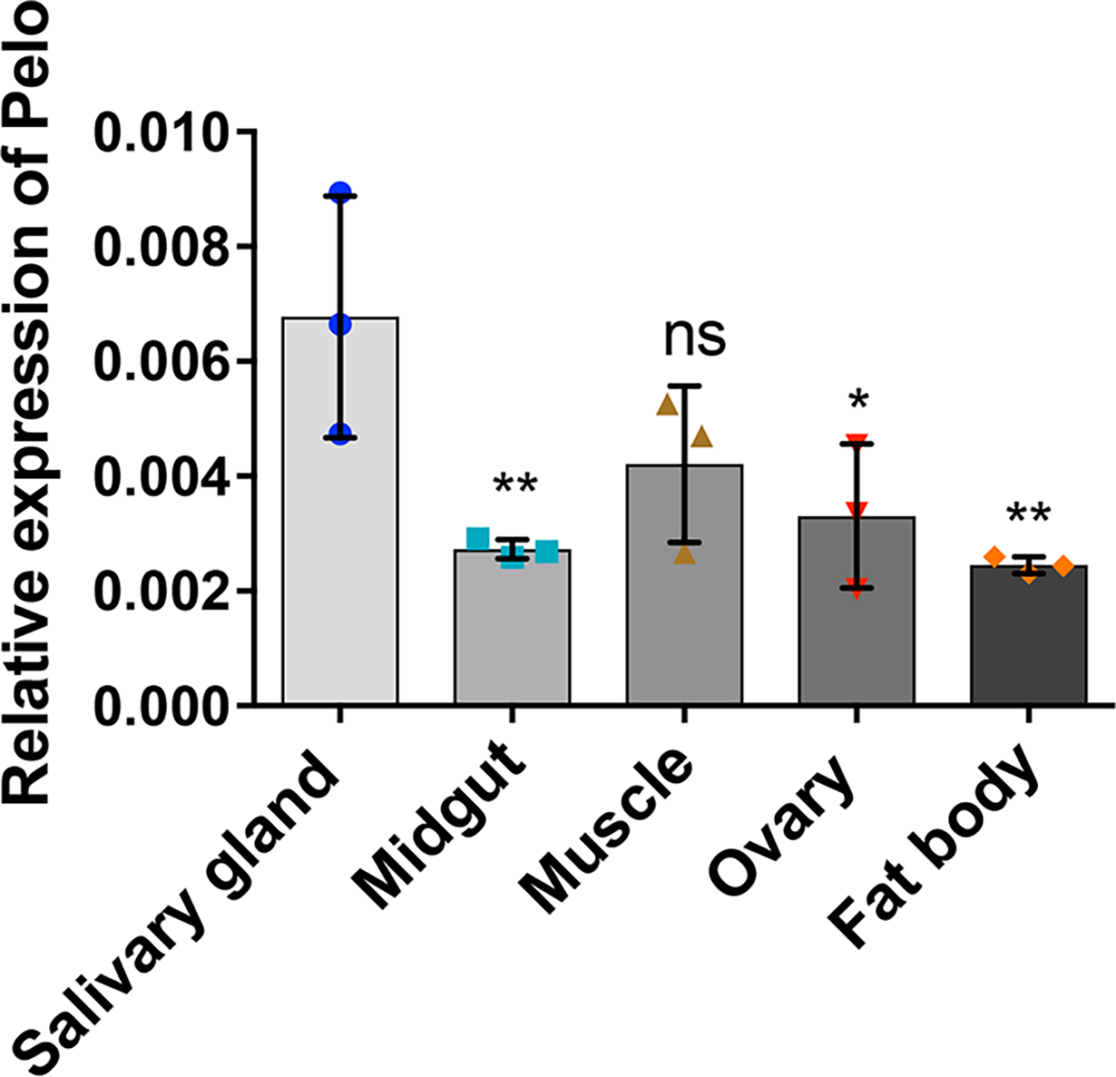
Tissue-specific expression of *pelo* in *Ae*. *aegypti* mosquitoes. RT-qPCR analysis of *pelo* transcript levels in the salivary gland, midgut, muscle, ovary and fat body of 3-day-old female mosquitoes (five mosquitos per biological replicate). Error bars represent standard error of the mean (SEM) in three biological replicates. An ANOVA test was performed followed by Tukey test on all pairwise comparisons. *, p<0.05; **, p<0.01, ns, not significant. Note: asterisks refer only to the Tukey pairwise comparisons between salivary glands and each of the other tissues.

Pelo has been demonstrated to be ubiquitously expressed in human and *D*. *melanogaster* tissues [24, 31]. This protein is also a highly conserved protein in mammals [24], and using multiple sequence alignments for pelo sequences from different insect species we found that the protein is also highly conserved among insect species (S1 Fig) with a distinct nuclear localization signal (PRKRK), and shows structural similarities with human pelo (S2 Fig). In order to investigate the tissue-specific expression of *Ae*. *aegypti pelo*, we dissected various tissues from 3-day-old *Ae*. *aegypti* female mosquitoes, including salivary glands, midgut, muscles, ovaries, and fat body. RT-qPCR results demonstrated that *pelo* is ubiquitously expressed in all the *Ae*. *aegypti* tissues tested. One-way ANOVA showed statistically significant differences between the different tissue groups (*F*(4, 10) = 5.786, *p* = 0.0112). Further Tukey post hoc test showed that significant differences were found in the transcript levels of *pelo* between salivary gland and fat body (*p* = 0.006), midgut (*p* = 0.0091) and ovary (*p* = 0.025), but not muscle (*p* = 0.094) (Fig 1). The difference in the levels of *pelo* was not significant among other tissues.

### *Wolbachia* suppresses *pelo* in *Ae*. *aegypti*

**Fig 2:**
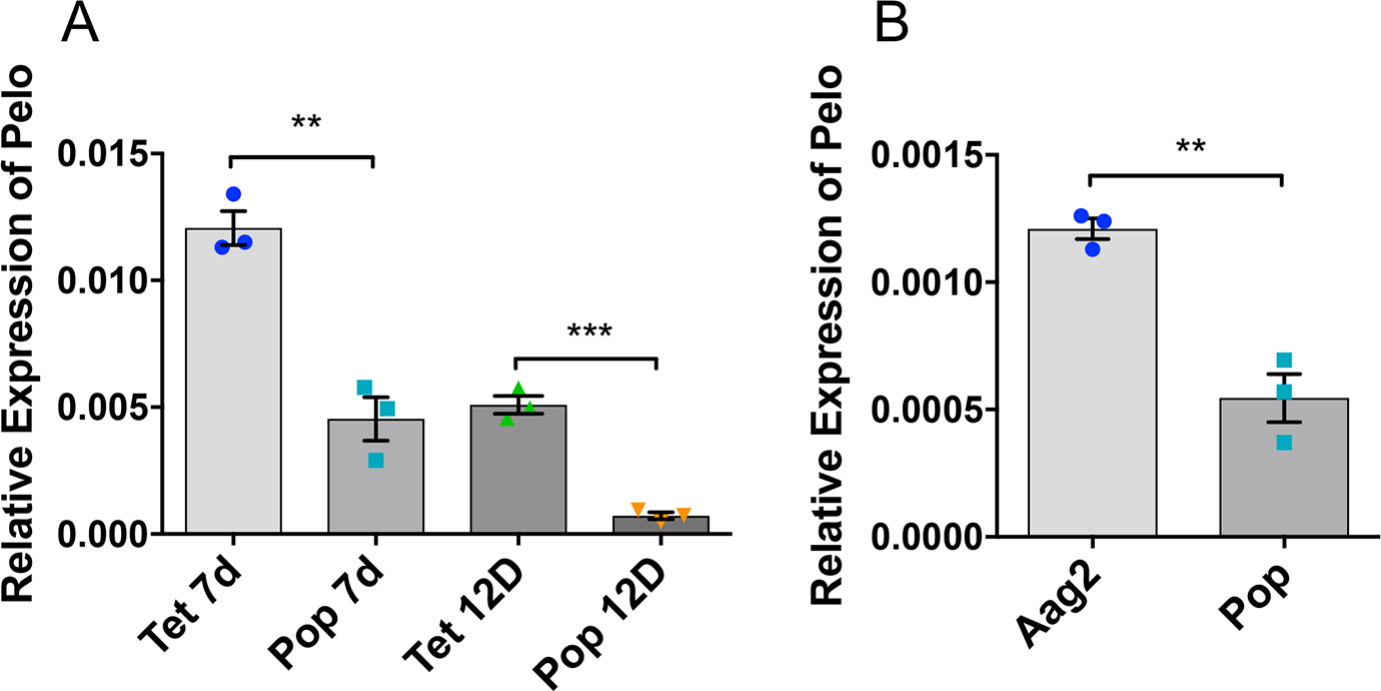
Relative expression of pelo in uninfected and *Wolbachia*-infected *Ae*. *aegypti* mosquitoes and cell lines. **A)** RT-qPCR analysis of *pelo* transcripts in 7-day- and 12-day-old female mosquitoes both uninfected (Tet) and infected (Pop) with *Wolbachia*. Error bars represent SEM from three biological replicates (**, p<0.01; ***, p<0.001; t-test). **B)** RT-qPCR quantification of the *pelo* transcript levels in uninfected (Aag2) and *Wolbachia-*infected (Pop) cell lines (**, p<0.01; t-test).

**Fig 3:**
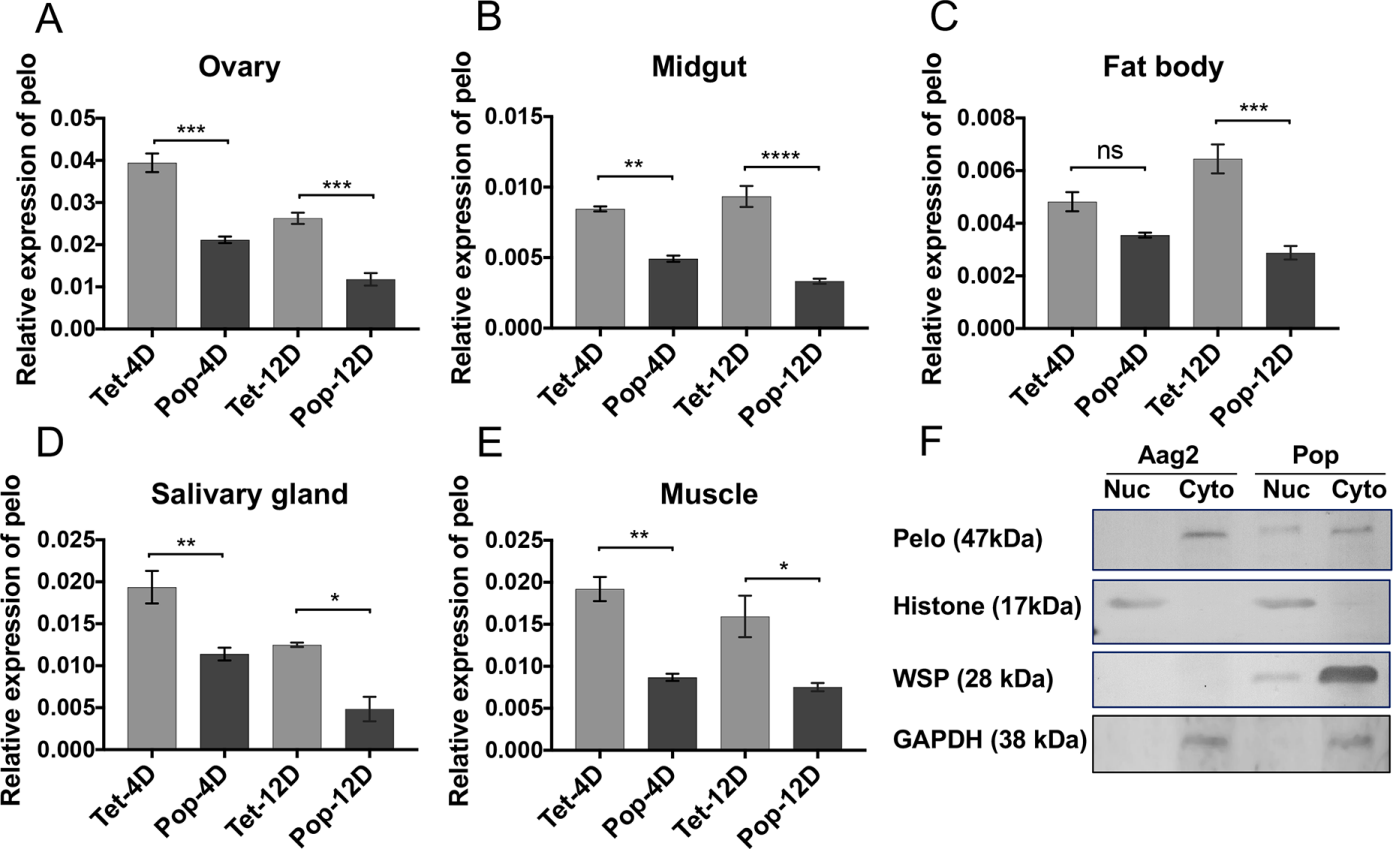
Tissue-specific modulation of pelo by *Wolbachia*. **A-E)** Relative expression of the *pelo* transcripts in different tissues of 4 and 12-day-old female mosquitoes (10 mosquitoes per biological replicate) in the absence (Tet) and presence of *Wolbachia* (Pop). **A)** ovary, **B)** midgut, **C)** fat body, **D)** salivary gland, and **E)** muscle. Error bars show SEM from three biological replicates each with two technical replications. **F)** Western blot analysis to examine the effect of *Wolbachia* on subcellular localization of the pelo protein showing translocation of pelo into the nucleus during *Wolbachia* infection. Anti-histone H3 and anti-GAPDH antibodies were used to check the efficiency of nuclear and cytoplasmic fractionations, while anti-WSP antibody was used to confirm the presence of *Wolbachia*. *, p<0.05; **, p<0.01; ***, p<0.001, ****, p<0.0001; ns, not significant; t-test.

In order to find the effect of *Wolbachia* on *pelo* gene expression, RT-qPCR was carried out using *pelo* gene-specific primers to examine its expression in tetracycline (Tet) treated and *Wolbachia w*MelPop-CLA infected (Pop) female mosquitoes at two different time points. Transcript levels of *pelo* were significantly reduced in Pop mosquitoes compared to Tet mosquitoes at 7-days (t = 6.932, df = 4, p<0.0023) and 12-days (t = 11.68, df = 4, p = 0.0003) post-emergence (Fig 2A). This result was also cross-validated in Aag2 and Aag2 cells infected with *w*MelPop-CLA (Pop) cells (Fig 2B; t = 6.495, df = 4, p = 0.0029). To further investigate in which tissue(s) *Wolbachia* affect *pelo*, relative expression levels of the gene were assessed in different tissues of both uninfected and infected female mosquitoes at 4 and 12-days after emergence by dissecting salivary gland, midgut, muscle, ovary and fat body from a total of 30 mosquitoes from each time point divided into three biological replicates. We found significant decreases in the *pelo* transcript levels in both 4-day (except for fat body in which a decrease trend was not significant) and 12-day-old Pop mosquitoes in ovary (*F*(3, 8) = 56.2, 4D p = 0.0001, 12D p = 0.0007), midgut (*F*(3, 8) = 49.14, p = 0.0013, p<0.0001), fat body (*F*(3, 8) = 19.41, p = 0.1316, p = 0.0005), salivary gland (*F*(3, 8) = 21.67, p = 0.009 and p = 0.012), and muscle (*F*(3, 8) = 14.88, p = 0.004, p = 0.015) tissues compared to those tissues of uninfected mosquitoes (Fig 3A–3E).

### *Wolbachia* affects the subcellular localization of the pelo protein

Pelo has been reported to be localized to the cytoplasm in *D*. *melanogaster* [32]. Although the pelo protein does have a conserved nuclear localization signal (PRKRK), there is a lack of experimental evidence of its presence inside the nucleus [24]; therefore, we explored its subcellular localization in *Ae*. *aegypti* cells and whether *Wolbachia* affects the localization of pelo. Nuclear and cytoplasmic fractions of both Aag2 and Pop cells were separated. Lysates were run on a 5–15% Bis/Tris polyacrylamide gel, total proteins were transferred to nitrocellulose membrane and then probed with an anti-pelo antiserum along with other control antisera, including anti-histone H3 (to confirm the nuclear fraction), anti-GAPDH (to confirm the cytoplasmic fraction), and anti-WSP (to confirm *Wolbachia* infection). Here it is important to note that we detected the WSP protein in the nuclear fraction of Pop cells as well, although in smaller amount as compared to the cytoplasmic fraction, which is consistent with the earlier report demonstrating the presence of *Wolbachia* (*w*MelPop) in the nuclei of *D*. *melanogaster* cells with the help of electron microscopy [14]. Nevertheless, the control antibodies showed a very good fractionation of the two cellular compartments. Our results demonstrated that in Aag2 uninfected cells, pelo was only detectable in the cytoplasmic fraction, however, *Wolbachia* infection led to the nuclear import of the protein thus affecting its subcellular localization as compared to uninfected Aag2 cells (Fig 3F). Further investigation is required to elucidate if the pelo protein plays any role inside the nucleus of *Wolbachia*-infected cells.

### *Wolbachia*-mediated downregulation of pelo is highly specific to female mosquitoes

**Fig 4:**
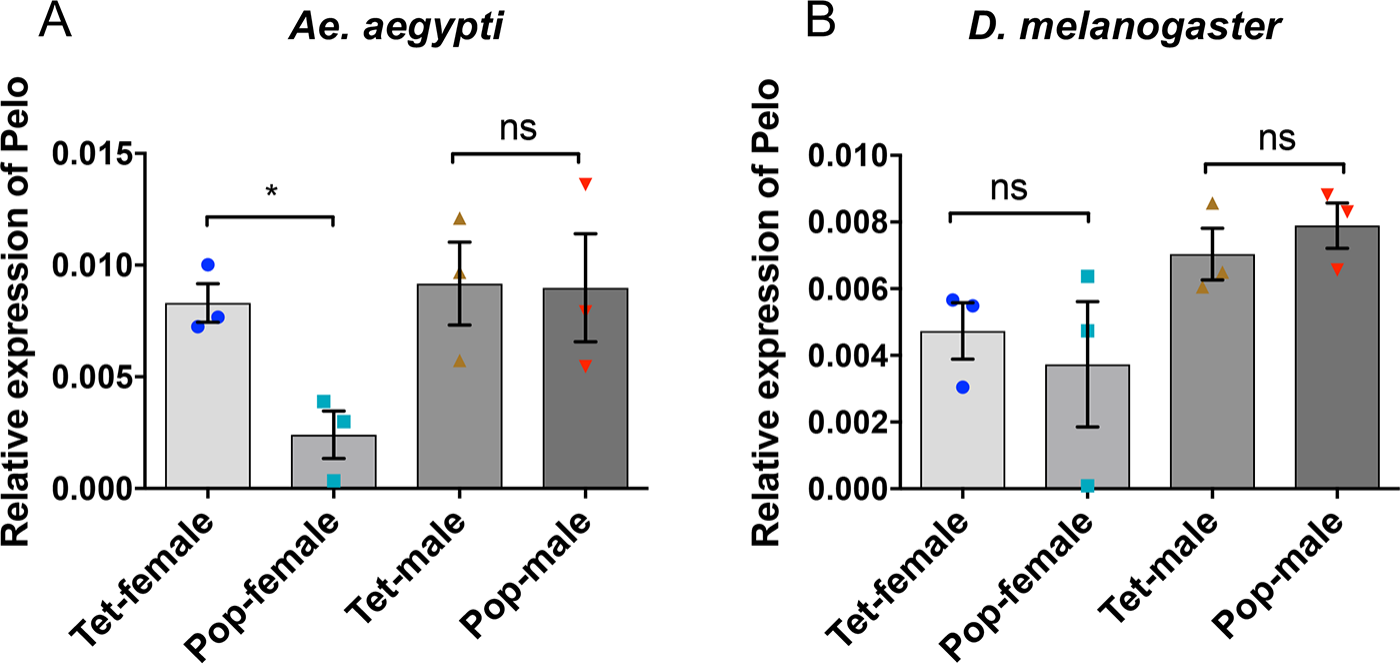
Effect of *Wolbachia* infection on the transcript levels of the *pelo* gene in male and female mosquitoes and flies. **A)** Relative expression of the *pelo* gene to *RPS17* housekeeping gene in 4-day-old female and male mosquitoes, both uninfected (Tet) and infected with *Wolbachia* (Pop) (*, p<0.05; t-test). **B)** Relative expression of the *pelo* gene to *RPS17* housekeeping gene in uninfected (Tet) and *Wolbachia-*infected (Pop) flies. Error bars represent SEM from three biological replicates (ns, not significant; t-test).

We measured the levels of the *pelo* transcripts in both male and female 4-day-old Tet and Pop mosquitoes. RT-qPCR analysis showed that there was no significant change in the levels of *pelo* between male Tet and Pop mosquitoes (Fig 4A; t = 0.06021, df = 4, p = 0.9549). Conversely, the level of *pelo* transcripts was significantly reduced in females in the presence of *Wolbachia* (Fig 4A; t = 4.307, df = 4, p = 0.0126). Although there is no evidence in *Ae*. *aegypti* mosquitoes to show differential host response to bacterial infections in the two sexes, a study carried out in the parasitoid *Asobara tabida* has highlighted differential immune response in males as compared to females challenged with *Wolbachia* strains *w*Atab1, *w*Atab2, and *w*Atab3, where males showed higher levels of immune gene expression upon *Wolbachia* infection as compared to females [33]. Our results also point towards a sex-specific response in *Ae*. *aegypti* mosquitoes during *Wolbachia* infection.

As *Wolbachia* is not a natural symbiont of *Ae*. *aegypti*, we were interested to test whether *Wolbachia* has the same suppressive effect on pelo in its natural host *D*. *melanogaster*. By measuring the relative levels of the *pelo* transcripts in Tet and *Wolbachia w*MelPop infected 4-day-old flies, we found that there was no significant change in the levels of *pelo* in infected and uninfected male and female flies (Fig 4B). The finding that the *Wolbachia w*MelPop-CLA infection suppresses *pelo* in females of the recently transinfected mosquito species *Ae*. *aegypti*, and not in its natural host *D*. *melanogaster* suggests that this effect could be attributed to the relatively recent association between *Wolbachia* and a new host.

### microRNA aae-miR-2940-5p is involved in regulation of pelo

**Fig 5:**
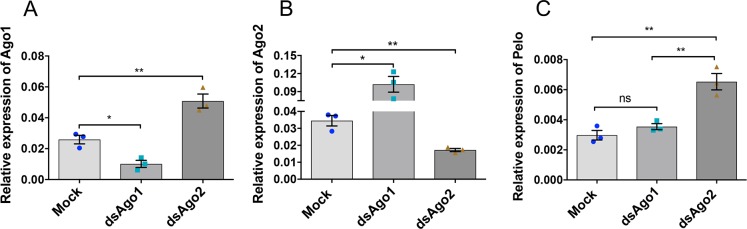
miRNA-mediated regulation of *pelo*. **A-B)** RT-qPCR analysis showing dsRNA-mediated silencing of *Ago1* (*, p<0.05; t-test) and *Ago2* (**, p<0.01; t-test) in Pop cells relative to mock, respectively. **C)** Relative expression of the *pelo* transcripts in *Ago1* and *Ago2* silenced Aag2 cells by RT-qPCR. Each error bar shows SEM from three biological replicates (**, p<0.01; ns, not significant; One-way ANOVA).

microRNAs (miRNA) are known to regulate different target genes. To find out whether the suppression of pelo in *Wolbachia*-infected mosquitoes is mediated by miRNA, we knocked down *Ago1* and *Ago2* genes in Pop cells, as both are important components of the RISC complex involved in miRNA functions [34, 35]. After confirming silencing of the Agos with dsRNA specific to the genes (Fig 5A and 5B), we employed RT-qPCR followed by the statistical analysis with One-way ANOVA (*F*(2, 6) = 25.1, *p* = 0.0012). Tukey post hoc test results showed that after *Ago2* knockdown there was a significant increase in the expression of *pelo* as compared to both mock (*p* = 0.0014) and *Ago1* knock down (*p* = 0.0035) (Fig 5C;), which suggested that miRNAs might be involved in regulating pelo levels. Interestingly, we found that when *Ago1* was silenced, *Ago2* levels increased (Fig 5A), and conversely when *Ago2* was knocked down, *Ago1* transcript levels increased (Fig 5B). This is consistent with previous studies carried out in *D*. *melanogaster* that Ago proteins may compensate for each other [36].

**Fig 6:**
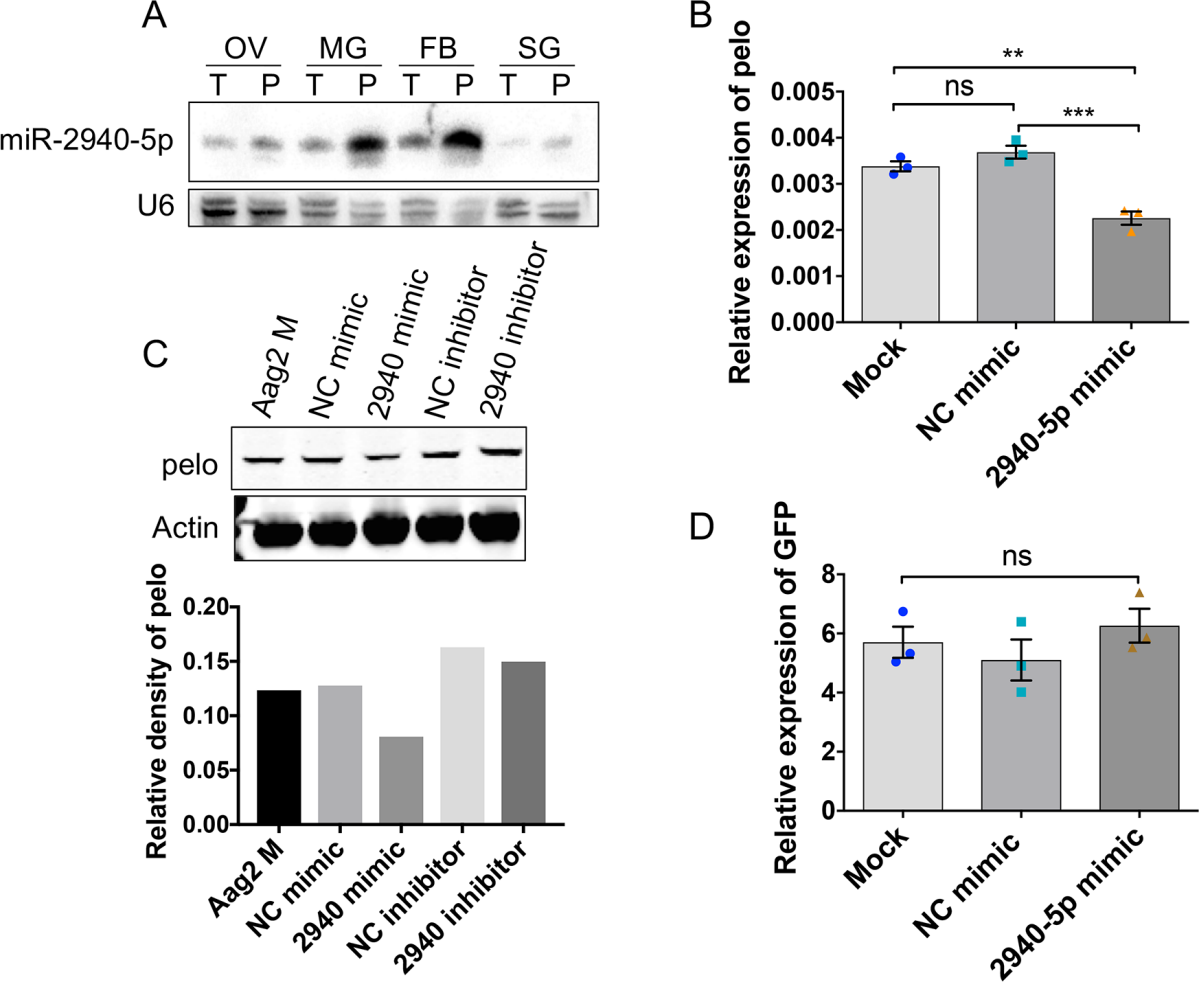
aae-miR-2940-5p dependent regulation of pelo. **A)** Northern blot analysis of RNA isolated from various tissues (OV, ovary; MG, midgut; FB, fat body; SG, salivary gland) of *w*MelPop (P) infected and tetracycline-treated (T) *Ae*. *aegypti* mosquitoes hybridized with a specific probe to aae-miR-2940-5p probe. U6 is shown as control. **B)** RT-qPCR performed to confirm the effect of oversupply of aae-miR-2940-5p mimic on *pelo* transcript levels. NC, negative control mimic with random sequence; Mock, no mimic with transfection reagent only. Error bars represent SEM from three biological replicates (**, p<0.01; ***, p<0.001). **C)** Western blot analysis to examine the effect of aae-miR-2940-5p mimic and inhibitor on pelo protein levels using an anti-pelo specific antibody (upper panel), while anti-beta-actin antibody was used as control to confirm equal loading of samples. Aag2 M, mock transfection; NC mimic, negative control mimic; 2940 mimic, aae-miR-2940-5p mimic; NC inhibitor, negative control inhibitor; 2940 inhibitor, aae-miR-2940-5p inhibitor. The relative density of pelo to actin determined by densitometric analysis using Image J is shown in the lower panel. **D)** RT-qPCR analysis of the effect of aae-miR-2940-5p mimic on the level of *GFP* transcripts in Sf9 cells transfected with pIZ/GFP fused with the pelo target sequences along with the negative control (NC) or aae-miR-2940-5p mimic. Mock, no mimic with transfection reagent only. ns, not significant; One-way ANOVA.

As *Wolbachia*-mediated pelo suppression was found to be specific to female *Ae*. *aegypti* mosquitoes, we aimed to determine if a mosquito-specific miRNA that was previously shown to be significantly up-regulated in *Wolbachia w*MelPop-CLA infected female mosquitoes, as compared to uninfected ones, could be involved in regulation of pelo [18]. Although three targets of this miRNA have already been identified [18, 19, 37], we tried to determine if it has any interaction with the transcripts of the *pelo* gene. RNAhybrid analysis showed potential aae-miR-2940-5p target sequences in the ORF of the *pelo* gene at positions 561–582 with significant seed region complementarity and high minimum free energy of -25.8 kcal/mol (S3 Fig). In order for a miRNA to regulate a target mRNA, it is important that the mature miRNA and its target transcript co-localize in the same tissue. As we found that *pelo* was expressed in all the mosquito tissues that we examined and *Wolbachia* supresses its expression in all the tested tissues (Fig 3A–3E), we were interested to find out the tissue-specific expression of aae-miR-2940-5p in mosquito tissues in the presence and absence of *Wolbachia*. Northern blot analysis of RNA isolated from various tissues (muscle was not included) of Pop and Tet *Ae*. *aegypti* mosquitoes confirmed induction of aae-miR-2940-5p due to *Wolbachia* infection in all tissues, with substantial induction in the midgut and fat body (Fig 6A). The trend in these inductions was of an inverse association with reductions of the *pelo* transcript levels in the corresponding tissues examined (Fig 3A–3D).

To verify the interaction between aae-miR2940-5p and *pelo*, Aag2 cells were transfected with a synthetic aae-miR2940-5p mimic, control mimic with random sequences, and without a mimic (mock transfection) as a second control. RT-qPCR results subjected to One-way ANOVA (*F*(2, 6) = 33, *p* = 0.0006) followed by Tukey post hoc test showed that there were significant reductions in the transcript levels of *pelo* in Aag2 cells transfected with aae-miR2940-5p mimic as compared to mock (*p* = 0.0022), and control mimic transfected cells (*p* = 0.0006) (Fig 6B). Furthermore, the effect of synthetic mimic and inhibitor of aae-miR-2940-5p on Pelo protein expression was analysed 72 h post-transfection. Western blot results suggested down-regulation of the pelo protein in the presence of the aae-miR-2940-5p mimic, while application of the inhibitor did not affect pelo levels as compared to the negative control inhibitor; it showed an increase when compared to the mock transfection or negative control mimic (Fig 6C).

In order to examine if there is direct interaction between aae-miR-2940-5p and the predicted target sequences in *pelo*, we cloned the target sequences downstream of *GFP* in the pIZ/V5-His vector. The *GFP*-*pelo* target sequence clone was co-transfected with the control random sequence mimic and aae-miR-2940-5p mimic into Aag2 cells. RNA was collected after 72 h, and RT-qPCR was carried out to check the level of *GFP* transcripts. The results showed that there was no change in the levels of *GFP* in response to aae-miR-2940-5p mimic (Fig 6D; *F*(2, 6) = 0.9271, *p* = 0.4458), which suggests that likely there is no direct interaction between *pelo* and aae-miR-2940-5p. This result is not surprising as most miRNAs are reported to target transcription factors or other proteins that in return fine tune the abundance of different transcripts [38]. To rule out the possibility of another target site for the miRNA in the *pelo* transcript undetected by the prediction program, the full-length *pelo*, including its 3’UTR, was cloned into the pIZ/V5-His vector and co-transfected with aae-miR-2940-5p mimic in Sf9 cells. There was no downregulation of *pelo* in the presence of aae-miR-2940-5p mimic (S4 Fig), which confirmed our aforementioned observation.

### Pelo plays a role in DENV virion production

**Fig 7:**
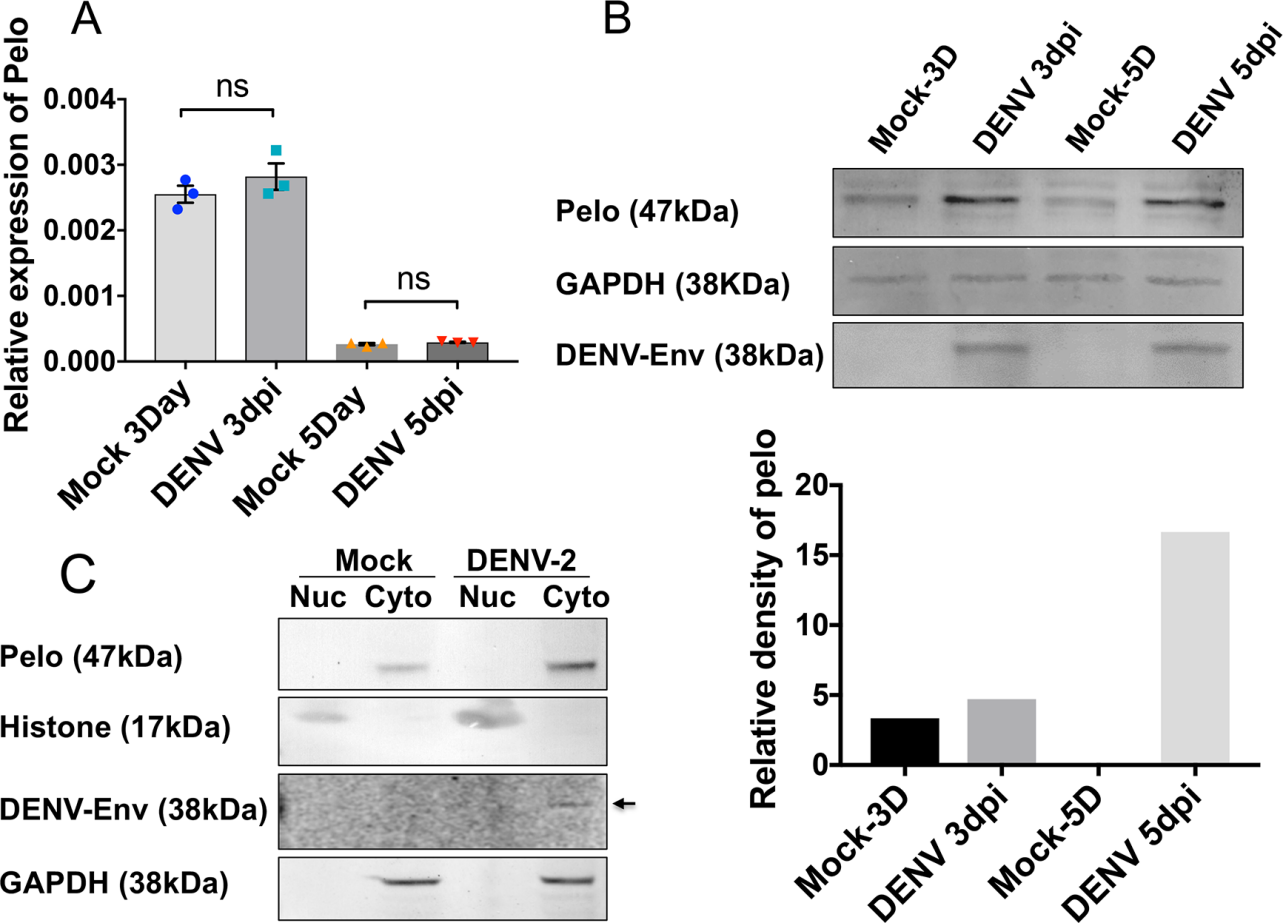
Expression pattern of pelo in DENV challenged mosquito cells. **A)** RT-qPCR analysis of *pelo* transcripts in the presence and absence of DENV challenge (1 MOI) in Aa20 cells at 3 and 5 dpi. Data was normalised using *RPS17* as the control gene. Error bars represent SEM from three biological replicates (ns shows no significance; t-test). **B)** Western blot analysis carried out using the anti-pelo specific antibody to check the pelo protein levels during DENV infection at 3 and 5 days post-infection (dpi), respectively. The relative density of pelo to actin determined by densitometric analysis using Image J is shown in the lower panel. **C)** Western blot results showing cellular localization of the pelo protein at 5 dpi with DENV. Histone H3 detection only in the nuclear fractions and GAPDH detection only in the cytoplasmic fractions show the success of subcellular fractionations, while DENV envelope protein only in the infected cells confirms DENV infection.

Recently, it has been reported that *pelo* is required for efficient replication of Drosophila C virus (DCV) [23]. In order to examine if the pelo protein has a similar effect on DENV replication, we infected *Ae*. *aegypti* Aa20 cells with DENV-2 (NGC strain) at 1 multiplicity of infection (MOI; pfu/cell). Cells were collected at 3 and 5 days post-infection (dpi) from which RNA and proteins were extracted. RT-qPCR results showed no significant change in the *pelo* transcript levels during DENV infection at both 3 (t = 1.12, df = 4, p = 0.3254) and 5 dpi (t = 1.447, df = 4, p = 0.2214) (Fig 7A). However, western blot analysis of the cells using an antiserum against *D*. *melanogaster* pelo clearly showed an increase in the levels of the protein upon infection at both 3 and 5 dpi (Fig 7B). The same blot was re-probed with an anti-DENV-Envelope antiserum to confirm infection. This suggests that pelo could be regulated at the post-transcriptional level. To check the localization of the pelo protein during DENV infection, we fractionated mock and 5 dpi Aa20 cells (1 MOI) into nuclear and cytoplasmic fractions. Both nuclear and cytoplasmic lysates were run on a 5–15% Bis/Tris polyacrylamide gel. After transfer to nitrocellulose membrane, the blot was probed with the pelo antiserum. Results showed that unlike *Wolbachia*, DENV infection has no effect on the subcellular localization of pelo, which remains in the cytoplasm in both uninfected and DENV-2 infected cells (Fig 7C).

**Fig 8:**
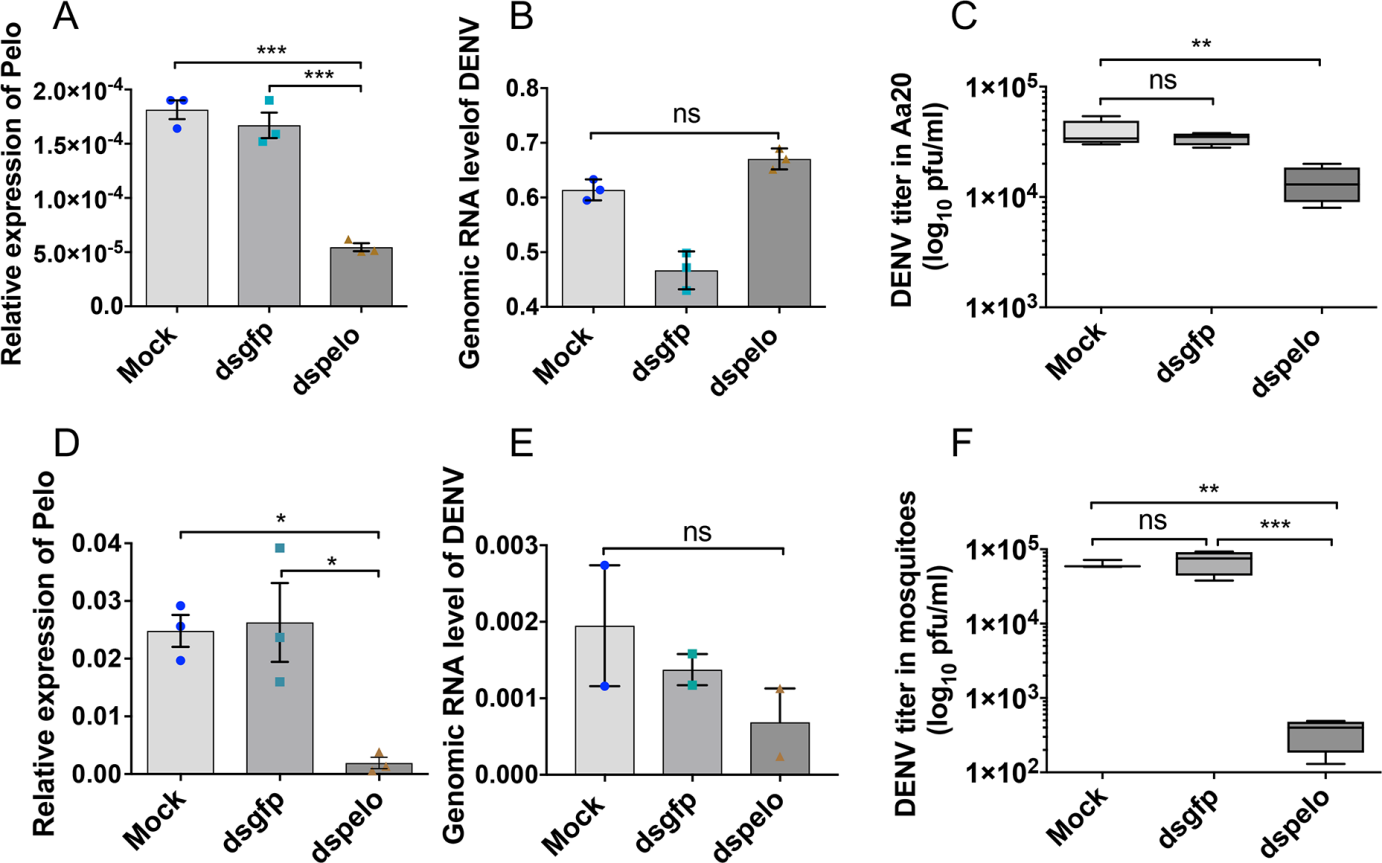
Silencing of *pelo* affects DENV virion production. **A)** RT-qPCR based confirmation of knock down of *pelo* in Aa20 cells. **B)** RT-qPCR analysis to examine the genomic RNA level of DENV in 3-day infected Aa20 cells (1 MOI) transfected with either no dsRNA (Mock) or with dsRNA against *GFP* as control or with dsRNA against *pelo* to silence the gene. *RPS17* was used to normalize the qPCR data, showing no effect at the DENV genomic RNA level. **C)** Viral titre determination by plaque assay conducted on the media collected from cells treated as in B. **D)** RT-qPCR based confirmation of knock down of *pelo* in *Ae*. *aegypti* mosquitoes. **E)** RT-qPCR analysis to examine the genomic RNA level of DENV in 7-day infected mosquitoes injected with either no dsRNA (Mock) or with dsRNA against *GFP* as control or with dsRNA against *pelo* to silence the gene. *RPS17* was used to normalize the qPCR data. **F)** Viral titre determination by plaque assay conducted on mosquito lysates prepared from mosquitoes treated as in E. Error bars represent SEM from three biological replicates. *, p<0.05; **, p<0.01; ***; ns, not significant; One-way ANOVA. Mock, no dsRNA and transfection reagent only; dsgfp, dsRNA to *green fluorescent protein* gene, dspelo, dsRNA to the *pelo* gene.

The above results suggest that DENV might increase levels of the host pelo protein in order to facilitate its replication, similar to DCV in *D*. *melanogaster*. In order to further confirm this link, the transcript levels of the *pelo* gene was knocked down in Aa20 cells using *pelo* specific dsRNAs (Fig 8A; about 70% silencing) and subsequently infected with DENV-2 (NGC strain) at 1 MOI. We carried out DENV infection experiments in *Ae*. *aegypti* cell line Aa20, because Aag2 cell line is persistently infected with insect-specific flavivirus cell fusing agent virus (CFAV) [39], but not Aa20 cells. At 3 dpi, cells were harvested and subjected to RT-qPCR using DENV-2 specific primers to the *NS1* gene. Surprisingly, RT-qPCR results showed no change in the DENV-2 genomic RNA levels in *pelo* depleted Aa20 cells as compared to mock and dsGFP transfected cells (Fig 8B; *F*(2, 6) = 3.106, p = 0.1186). However, plaque assay displayed a significant reduction (*F*(2, 9) = 12.88, *p* = 0.0023) in the virus titre in the medium collected from Aa20 cells treated with dspelo and infected with DENV-2 compared to both mock (*p* = 0.0028) and dsGFP (*p* = 0.0084) treated Aa20 cells followed by DENV-2 infection (Fig 8C). These results demonstrated that when *pelo* was knocked down the number of DENV infectious particles produced and secreted outside the cells significantly declined, but there was no effect on the total genomic RNA of DENV, implicating that pelo might play an important role in the translation of the viral genomic RNA and/or assembly and release of DENV-2 virions.

To show the effect of *pelo* silencing *in vivo*, we initially injected 4-day-old mosquitoes with dsPelo, dsGFP and PBS, and after two days fed them with sheep blood containing 10^7^ pfu/ml DENV-2. Seven days following virus inoculation, mosquitoes were collected and tested for DENV genomic RNA levels in the treatments using RT-qPCR. This time point was selected to allow sufficient time for the virus to replicate for comparison between the treatments. However, we detected very little viral RNA in the samples (S5 Fig), and further testing of individual mosquitoes determined that only about 13% of the mosquitoes were infected with DENV. Although oral inoculation is the natural route of infection, since our main purpose was to find out if dsPelo treatment has the same effect on DENV replication in mosquitoes as in the cell line, we also injected mosquitoes with virus, which resulted in 100% infection. Silencing *pelo* in mosquitoes when injected with dspelo (Fig 8D; about 92%) followed by DENV injection also showed no significant change (*F*(2, 3) = 4.719, *p* = 0.118) in DENV gRNA levels (Fig 8E), but significant reduction (*F*(2, 8) = 22.74, *p* = 0.0005) in DENV titres as compared to mock (*p* = 0.0022) or dsGFP (*p* = 0.0006) injected mosquitoes at 7 dpi (Fig 8F). The sample size we used for the *in vivo* assay was not high, but it corroborated with the *in vitro* results.

## Discussion

Despite numerous efforts to unveil the mechanism by which *Wolbachia* manipulates its host environment to restrict virus replication, the exact mechanism(s) that govern this antiviral property are largely unknown. In this study, we have found that the *Wolbachia* supresses the pelo protein which in turn may contribute towards restricting DENV virion production in *Ae*. *aegypti*.

*Wolbachia* is a facultative endosymbiont in many insect species and other invertebrates, however, the *w*MelPop-CLA strain, which most effectively suppresses DENV replication, has no natural association with the primary DENV vector *Ae*. *aegypti* and has been artificially transinfected into *Ae*. *aegypti* [13]. Several reports have shown that *Wolbachia*-mediated activation of immune genes in the Imd and Toll pathways could be involved in host protection from various viruses in mosquitoes [11, 40, 41]. Other reports, however, have shown that *Wolbachia* does not elicit the host immune response, in particular in hosts that are naturally infected with *Wolbachia* [42–44]. A recent study investigating the role of *Wolbachia*-induced restriction of Semliki Forest virus within *D*. *melanogaster* cells (JW18) suggested that interference occurs at a very early stage of infection and at the level of viral RNA translation or host RNA transcription [45]. However, in some *Wolbachia*-host-virus associations viral replication is not inhibited but rather a tolerance to viral infection is conferred by *Wolbachia* [46, 47].

Recently, findings of Wu et al. (2014) and Lapidopt et al. (2015) have shed light on the role of the pelo protein as an important host factor for effective viral replication in the case of Drosophila C virus (DCV) and Tomato yellow leaf curl virus (TYLCV) [23, 48]. However, the potential role of pelo has not been characterized yet in the case of the medically important DENV and its controlling agent *Wolbachia* in the mosquito vector. RT-qPCR analyses revealed that the *pelo* gene is expressed ubiquitously throughout all the main tissues of *Ae*. *aegypti* with higher transcript levels in the salivary glands, which is consistent with the studies conducted on tissue localization of pelo in the case of human and *D*. *melanogaster* [24, 31]. Interestingly, we found that *pelo* is supressed in the presence of *Wolbachia* in cell lines, whole mosquitoes and a number of tissues such as the salivary gland, muscle, and ovary. However, this suppression is specific to female mosquitoes; it was not seen in the male mosquitoes or in either male or females of *D*. *melanogaster* infected with *w*MelPop. The difference in pelo regulation in *Wolbachia*-infected *Ae*. *aegypti* versus *D*. *melanogaster* could be due to the length of association between the bacteria and host; *Wolbachia* is a natural infection in the fly whereas its infection of *Ae*. *aegypti* mosquitoes was only recently established through transinfection [49].

DENV titre in Pop cells was below the detection limit of our plaque assay, and others [11, 12] have also shown that barely any virion is produced in *w*MelPop-infected cells or mosquitoes that is detectable by plaque assay. While in the Aa20 cell line treated with dspelo, inhibition of DENV was not as dramatic as seen in *w*MelPop-infected mosquitoes or the cell line, silencing the gene in mosquitoes had very similar effects on DENV replication to *w*MelPop-infected mosquitoes, which could be due to higher efficiency of gene silencing. Nevertheless, as indicated above, downregulation of pelo in the presence of *Wolbachia* does not seem to be the only mechanism of virus blocking.

Pelo is a highly conserved protein in mammals [24], and using multiple sequence alignments for pelo sequences from different insect species we found that the protein is also highly conserved among insect species (S1 Fig). It has a distinct nuclear localization signal (PRKRK), and shows structural similarities with human pelo (S2 Fig). However, it has been demonstrated that pelo mostly resides in the cytoplasm and there is a lack of evidence for the presence of pelo in the nucleus [32]. This led us to investigate the localization of the pelo protein in mosquito cells and in particular in the instance of *Wolbachia* infection. Western blot results showed that the pelo protein is mainly found in the cytoplasm of mosquito cells when uninfected, however, *Wolbachia* infection leads to a change in the subcellular localization of pelo, which was found both in the cytoplasm and the nucleus. This change in the subcellular localization of pelo during *Wolbachia* infection may restrict availability of the protein in the cytoplasm, which is required for rapid translation of specific viral proteins. This would be in agreement with a previous study in which it was shown that pelo is required for the synthesis of the capsid protein of DCV, which they described as a quickly translated protein [23]. Alternatively, the translocation of pelo into the nucleus may have an inhibitory effect on a host gene(s) that normally facilitates viral replication.

miRNAs are important regulators of different cellular processes including cell division, timing, differentiation, death, control of metabolism, transposon silencing and antiviral defence [50–52]. Recently, our group has shed light on the modulation of different cellular miRNAs in the case of infection with *w*MelPop strain of *Wolbachia* in female mosquitoes [18]. Along similar lines, in this study we have investigated whether pelo has an interaction with any miRNA by silencing both *Argonaute 1* (*Ago1*) and *Argonaute 2* (*Ago2*) genes, which are important components of the RISC complex and involved in miRNA function [36, 53]. Interestingly, we found that silencing of one Ago led to the upregulation of the other Ago. One possible reason might be class switching between the Agos [36]. Increase in *pelo* levels in *Ago2* silenced cells suggested that *pelo* downregulation in *Wolbachia*-infected cells could possibly be mediated by miRNA. Hussain et al (2011) provided evidence that in *Wolbachia w*MelPop-CLA infected mosquitoes the mosquito-specific aae-miR-2940-5p was significantly upregulated as compared to uninfected mosquitoes [18]. While three targets of this miRNA have already been identified [18, 19, 37], we explored whether it has any interaction with the transcripts of the *pelo* gene. The co-localization of both *pelo* transcripts and mature aae-miR-2940-5p highlights the possibility that in *Wolbachia*-infected cells aae-miR-2940-5p might be utilized to downregulate *pelo* transcripts. These results also further confirmed differential expression of this miRNA in *Wolbachia*-infected mosquitoes at the tissue level. Further investigation showed that *pelo* transcript levels were downregulated in the presence of the artificially synthesized mimic of aae-miR-2940-5p both at the transcript and the protein levels, while there was an increase in the pelo protein in the case of addition of an artificially synthesized inhibitor of aae-miR-2940-5p. However, target validation results using *GFP* as a reporter suggested that *pelo* may not be a direct target of aae-miR-2940. The action of aae-miR-2940-5p could be through regulation of a transcription factor(s) or other protein(s) by the miRNA that fine-tunes the abundance of the *pelo* transcript [38].

Viruses are well known to modulate transcripts of host cells for their own benefit. Recently, a research group examined *D*. *melanogaster* mutants that resist DCV replication through a forward genetic screen and demonstrated a DCV-resistant mutant to be deficient of the *pelo* gene [23]. Pelo protein has also been implicated in TYLCV resistance in tomato TY172 cultivar [48]. However, its potential role in the case of DENV has not yet been explored. Our results suggest an increase in the levels of pelo in DENV-infected cells at the protein level without a change in its subcellular localization, which was mostly cytoplasmic. Furthermore, *pelo* knockdown studies suggest it plays a role in DENV virion production/secretion. The outcome of this study is in agreement with the previous findings that showed pelo is vital for the translation of the capsid protein of DCV thus positively affecting replication of DCV. A similar role of pelo was found in other viruses including Cricket Paralysis virus, Double Drosophila X virus, and invertebrate iridescent virus 6 and TYLCV replication [23, 48], suggesting that pelo might be an important host factor that is recruited by viruses to facilitate the translation of viral genome.

In summary, we have demonstrated that the pelo protein facilitates DENV production/secretion, and in female *Ae*. *aegypti* mosquitoes, *Wolbachia* suppresses the pelo protein, which may consequently contribute to restriction of DENV in the mosquitoes. This effect could be due to relocalization of the pelo protein to the nucleus in *Wolbachia*-infected cells as compared to non-infected cells. In addition, regulation of pelo in *Wolbachia*-infected mosquito cells appears to be mediated by aae-miR-2940-5p. However, the regulation of pelo was found to be female mosquito specific and not observed in male mosquitoes or in *D*. *melanogaster* males or females. Therefore, while suppression of pelo in female mosquitoes may contribute to virus inhibition, this does not seem to be the universal mechanism of virus inhibition seen across different host-virus interactions. However, identification of an association between pelo, the miRNA aae-miR-2940-5p and virus inhibition in *Wolbachia-*infected *Ae*. *aegypti* provides new insights into the mechanism of *Wolbachia* induced virus inhibition, which is a central feature of a major new strategy against arboviral diseases.

## Supporting information

S1 Fig Pelo is highly conserved among insects: Schematic representation of the level of conservation between different insect species listed in the S2 Table. The nuclear localization signal is boxed.
(TIF)

S2 Fig *Aedes aegypti* pelo is structurally similar to the human pelo. **A)** I-Tasser predicted model of *Ae*. *aegypti* pelo protein; **B)** Ramachandran plot showing more than 90% of residues fall in the allowed region thus confirming the strength of the model. **C)** Experimentally verified C-terminus model of human pelo. **D)** Superimposition of *Ae*. *aegypti* predicted pelo protein model and human pelo shows that they are highly similar in structure.
(TIF)

S3 Fig Sequence complementarity of aae-miR-2940-5p with its predicted target pelo. (TIF)

S4 Fig *Pelo* transcript is not a direct target of aae-miR-2940-5p. The full-length *pelo* transcript, including the 3’UTR was cloned into the pIZ vector and transfected into Aag2 cells either without or with negative control (NC) or aae-2940-5p mimic. The results confirmed overexpression of *pelo* in cells transfected with pIZ/Pelo only. Application of the aae-miR-2940-5p did not significantly change the expression levels of *pelo*, indicating there is no direct interaction of the miRNA with the transcripts of *pelo*. Error bars represent SEM from three biological replicates (**, p<0.01; ***, p<0.001; ns, not significant; One-way ANOVA).
(TIF)

S5 Fig Examining the effect of *pelo* silencing on DENV-2 replication in *Ae*. *aegypti* mosquitoes. 4-day-old female mosquitoes were injected with PBS (Mock), dsRNA to GFP or dsRNA to *pelo* and after 2 days fed with sheep blood containing 10^7^ pfu/ml DENV-2. RT-qPCR analysis of RNA extracted from the mosquitoes seven days after virus inoculation detected very little virus replication.
(TIF)

S1 Table Primers used in this study. (DOCX)

S2 Table Pelo protein sequences used for multiple sequence alignment. (DOCX)
