# Supplementary figures and images for: Suppression of the pelo protein by Wolbachia and its effect on dengue virus in Aedes aegypti
Source: PLoS Negl Trop Dis. 2018 Apr 11;12(4):e0006405. doi: 10.1371/journal.pntd.0006405 (PMC5912784; doi:10.1371/journal.pntd.0006405)

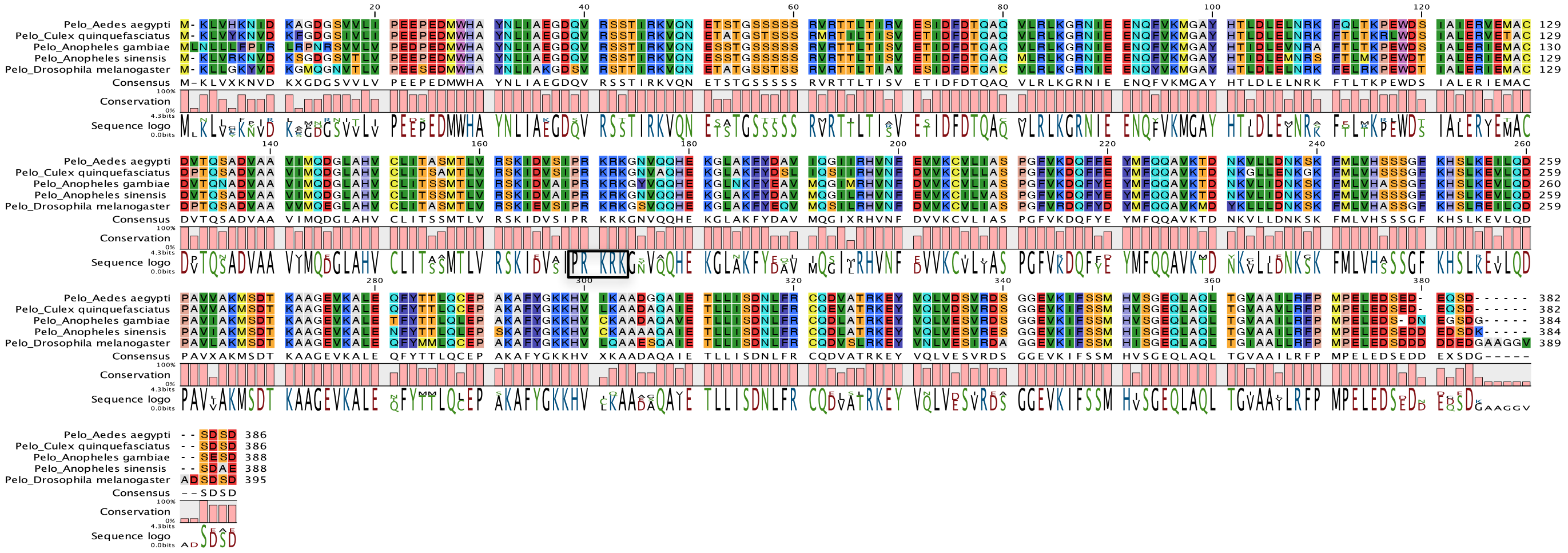

Supplement: S1 Fig — The nuclear localization signal is boxed. (TIF) [file pntd.0006405.s001.tif]

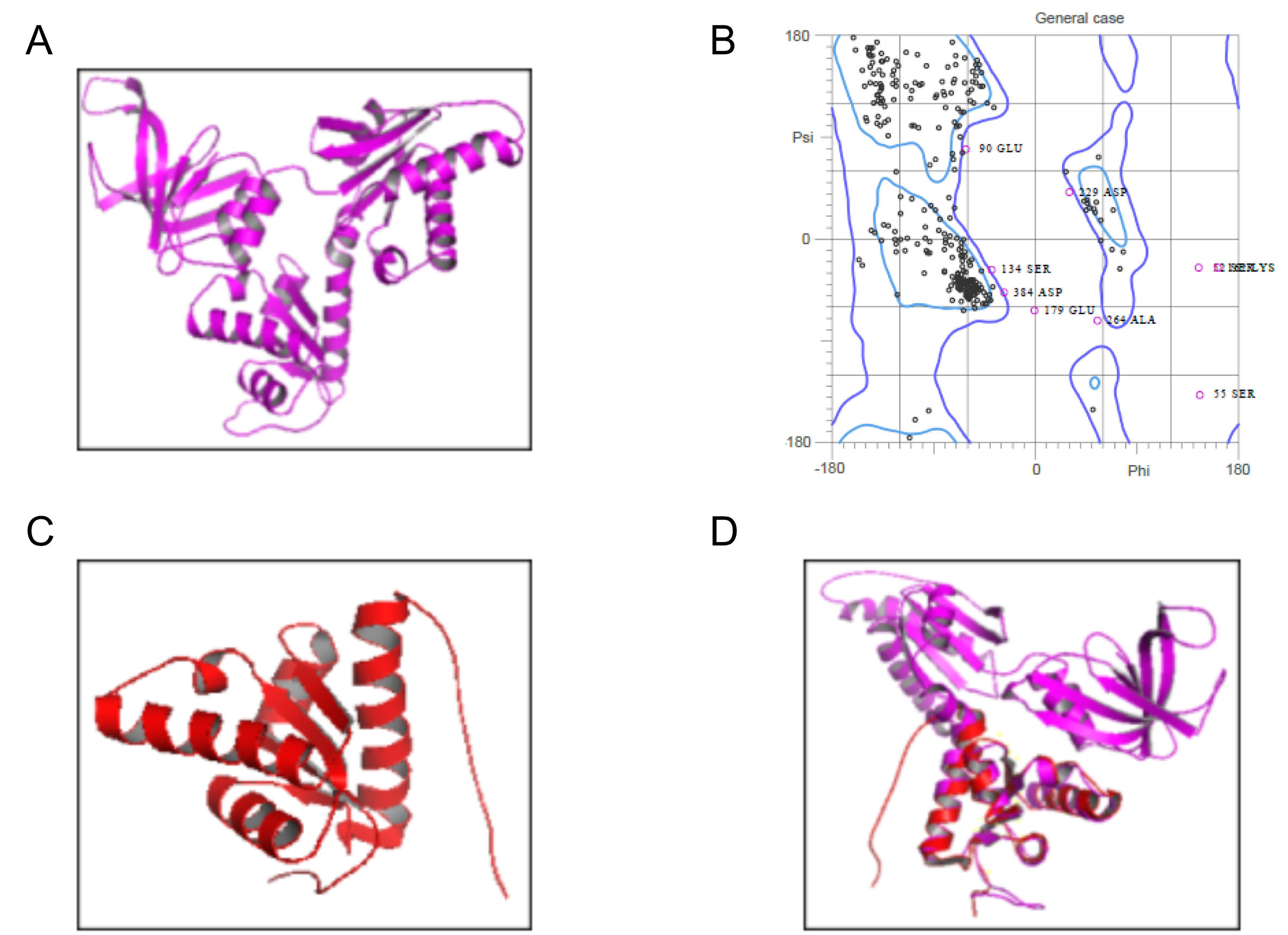

Supplement: S2 Fig — A) I-Tasser predicted model of Ae. aegypti pelo protein; B) Ramachandran plot showing more than 90% of residues fall in the allowed region thus confirming the strength of the model. C) Experimentally verified C-terminus model of human pelo. D) Superimposition of Ae. aegypti predicted pelo protein model and human pelo shows that they are highly similar in structure. (TIF) [file pntd.0006405.s002.tif]

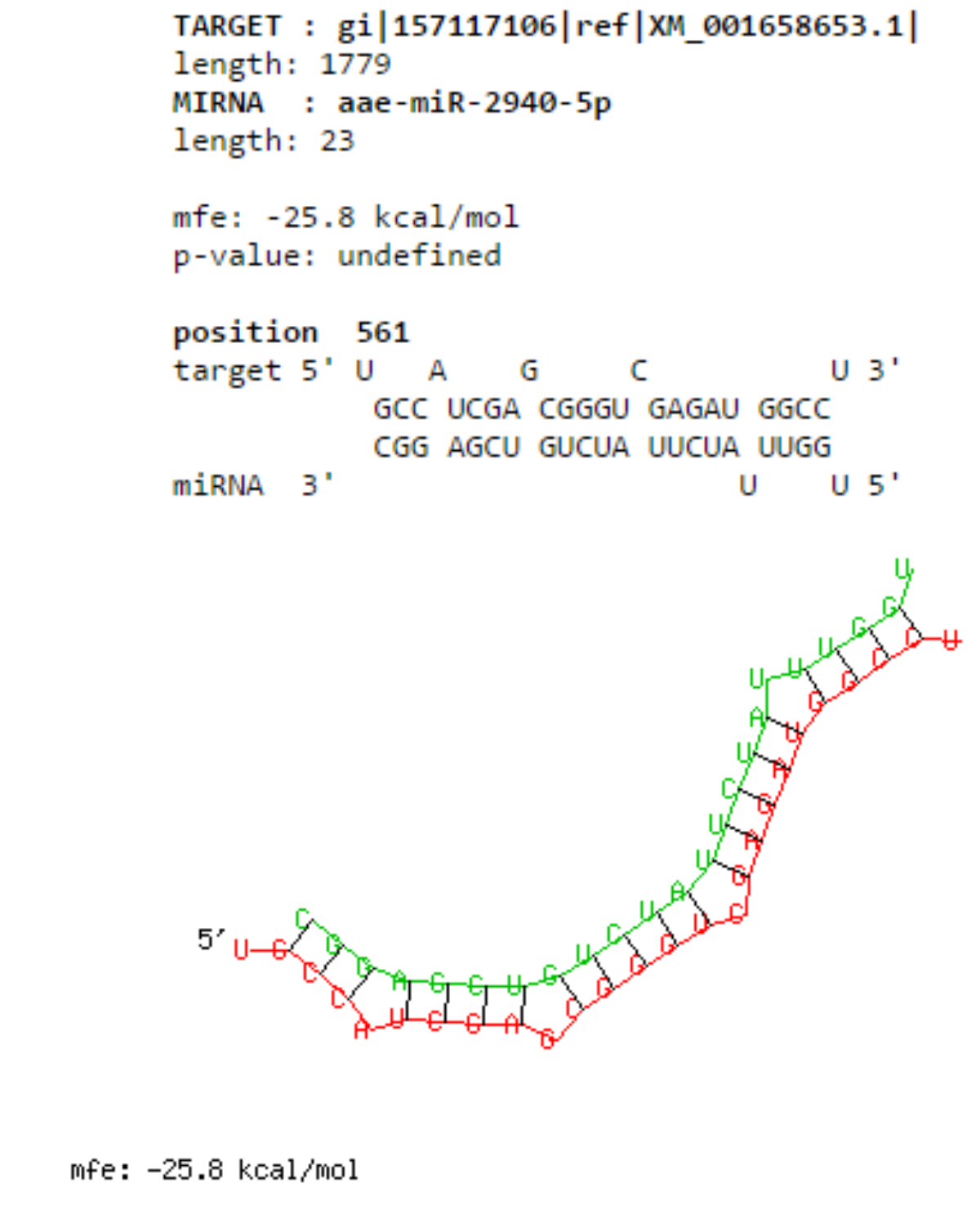

Supplement: S3 Fig — (TIF) [file pntd.0006405.s003.tif]

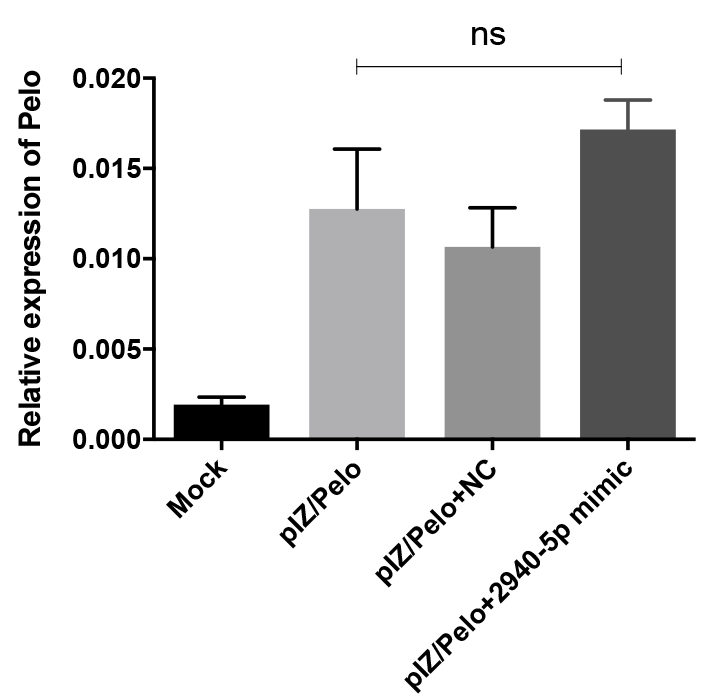

Supplement: S4 Fig — The full-length pelo transcript, including the 3’UTR was cloned into the pIZ vector and transfected into Aag2 cells either without or with negative control (NC) or aae-2940-5p mimic. The results confirmed overexpression of pelo in cells transfected with pIZ/Pelo only. Application of the aae-miR-2940-5p did not significantly change the expression levels of pelo, indicating there is no direct interaction of the miRNA with the transcripts of pelo. Error bars represent SEM from three biological replicates (**, p<0.01; ***, p<0.001; ns, not significant; One-way ANOVA). (TIF) [file pntd.0006405.s004.tif]

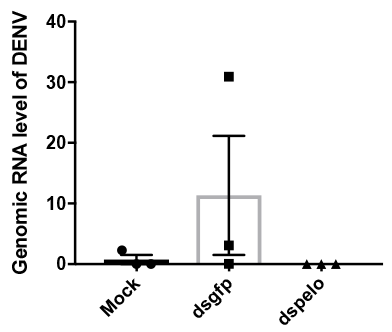

Supplement: S5 Fig — 4-day-old female mosquitoes were injected with PBS (Mock), dsRNA to GFP or dsRNA to pelo and after 2 days fed with sheep blood containing 107 pfu/ml DENV-2. RT-qPCR analysis of RNA extracted from the mosquitoes seven days after virus inoculation detected very little virus replication. (TIF) [file pntd.0006405.s005.tif]
