# Supplementary material for: Suppression of the pelo protein by Wolbachia and its effect on dengue virus in Aedes aegypti
Source: PLoS Negl Trop Dis. 2018 Apr 11;12(4):e0006405. doi: 10.1371/journal.pntd.0006405 (PMC5912784; doi:10.1371/journal.pntd.0006405)
Supplement: S1 Table — (DOCX) [file pntd.0006405.s006.docx]

**Table S1:** Primers used in this study.

| **Gene Name** | **Primer Sequence (5’-3’)** |
| --- | --- |
| Ae-pelo-qF | CAAGGCCTTCTACGGCAAGA |
| Ae-pelo-qR | GAATCACGCACCGAATCGAC |
| Ae-Ago1-qF | CGCAGACAAGAAGGAACAGA |
| Ae-Ago1-qR | TCCCACAGGACGTGATAATG |
| Ae-Ago2-qF | CTGGACATGACTTGCCTGAA |
| Ae-Ago2-qR | AGCTCATGGTTGCTTCCAAT |
| Ae-RPS17-qF | CACTCCGAGGTCCGTGGTAT |
| Ae-RPS17-qR | GGACACTTCGGGCACGTAGT |
| Dm-pelo-qF | CGAGCATGCACATCTCAGGA |
| Dm-pelo-qR | TCGCTATCGCTATCTGCCAC |
| Dm-RPL32-qF | GACGCTTCAAGGGACAGTATCTG |
| Dm-RPL32-qR | AAACGCGGTTCTGCATGAG |
| Ae-pelo-RNAi-F | TAATACGACTCACTATAGGGTCTTACCATCCGGGTCGAGT |
| Ae-pelo-RNAi-R | TAATACGACTCACTATAGGGCCAGACGACGAGTGAACCAA |
| Ae-pelo-full length-F | GGACTAGTATGAAGTTGGTTCATAAAAACATTG |
| Ae-pelo-full length-R | GGTCTAGATCAATCCGAGTCGGAATC |
| Ae-Ago1-RNAi-F | TAATACGACTCACTATAGGGCGCCAAGGCCTCCAAGTCCG |
| Ae-Ago1-RNAi-R | TAATACGACTCACTATAGGGATACCGGCTTCAGCGCACCG |
| Ae-Ago2-RNAi-F | TAATACGACTCACTATAGGGATGTAGACGCGTCCTCTGT |
| Ae-Ago2-RNAi-R | TAATACGACTCACTATAGGGACAGTTCAAGCAGACGAACC |
| DENV-qF | GGTATGGTGGGCGCTACTA |
| DENV-qR | CAAGGCTAACGCATCAGTCA |
