# Supplementary material for: Suppression of the pelo protein by Wolbachia and its effect on dengue virus in Aedes aegypti
Source: PLoS Negl Trop Dis. 2018 Apr 11;12(4):e0006405. doi: 10.1371/journal.pntd.0006405 (PMC5912784; doi:10.1371/journal.pntd.0006405)
Supplement: S2 Table — (DOCX) [file pntd.0006405.s007.docx]

**Table S2.** Pelo protein sequences used for multiple sequence alignment.

| Species Name | Uniprot ID | Identity | Score |
| --- | --- | --- | --- |
| *Aedes aegypti* | Q170N2 | 100 | 1,946 |
| *Drosophila melanogaster* | P48612 | 81.8 | 1,673 |
| *Culex quinquefasciatus* | B0WGL0 | 91.7 | 1,813 |
| *Anopheles gambiae* | Q7Q477 | 83.6 | 1,714 |
| *Anopheles sinensis* | A0A084W4V1 | 90.1 | 1,723 |
| *Homo sapiens* | Q9BRX2 | 66.1 | 1,359 |
